# Polystyrene nanoparticles induce concerted response of plant defense mechanisms in plant cells

**DOI:** 10.1038/s41598-023-50104-5

**Published:** 2023-12-16

**Authors:** Sylwia Adamczyk, Joanna Chojak-Koźniewska, Sylwia Oleszczuk, Krzysztof Michalski, Sannakajsa Velmala, Laura J. Zantis, Thijs Bosker, Janusz Zimny, Bartosz Adamczyk, Slawomir Sowa

**Affiliations:** 1 Natural Resources Institut Finland (Luke), Latokartanonkaari 9, 00790 Helsinki, Finland; 2 Plant Breeding and Acclimatization Institute - National Research Institute, Radzikow, 05-870 Blonie, Poland; 3 Institute of Environmental Sciences, Leiden University, P.O. Box 9518, 2300 RA Leiden, The Netherlands; 4 Leiden University College, Leiden University, P.O. Box 13228, 2501 EE The Hague, The Netherlands

**Keywords:** Abiotic, Chemical ecology, Plant signalling

## Abstract

Recent advances in knowledge suggest that micro- and nanoplastics pose a threat to plant health, however, the responses of plants to this stressor are not well-known. Here we examined the response of plant cell defence mechanisms to nanoparticles of commonly used plastic, polystyrene. We used plant cell cultures of widely cultivated plants, the monocots wheat and barley (*Triticum aestivum* L., *Hordeum vulgare* L.) and the dicots carrot and tomato (*Daucus carota* L., *Solanum lycopersicum* L.). We measured the activities of enzymes involved in the scavenging of reactive oxygen species and nonenzymatic antioxidants and we estimated potential damages in plant cell structures and functioning via lipid peroxidation and DNA methylation levels. Our results demonstrate that the mode of action of polystyrene nanoparticles on plant cells involves oxidative stress. However, the changes in plant defence mechanisms are dependent on plant species, exposure time and nanoplastic concentrations. In general, both monocots showed similar responses to nanoplastics, but the carrot followed more the response of monocots than a second dicot, a tomato. Higher H_2_O_2_, lipid peroxidation and lower enzyme activities scavenging H_2_O_2_ suggest that tomato cells may be more susceptible to polystyrene-induced stress. In conclusion, polystyrene nanoplastics induce oxidative stress and the response of the plant defense mechanisms involving several chain reactions leading to oxidoreductive homeostasis.

## Introduction

We live in a “plastic age”, as over 200 million tons of disposable plastic is utilized each year^1^. Plastics are an integral part of agricultural production as mulch films, nets, storage bins and many other applications^2^. In agriculture and horticulture, we commonly use polyethylene, polyethylene terephthalate, polypropylene and polystyrene^3^. Although it has been assumed that plastics are inert compounds, recent advancements in the knowledge revealed the potential toxic effect of plastics on living organisms^1^. Of specific concern are products of plastic degradation, namely micro- and nanoplastics (MNPs), having particle sizes of 5 mm-100 nm and < 100 nm, respectively^4^. The concentration of MNPs in agricultural soil may reach 4.5 mg kg^−1^^5^. However, the exact MNPs concentration in soil is not known as established procedures for their collection and characterization are lacking, thus the concentrations may be underestimated, especially for smaller particle sizes^6^. Most studies to date have concentrated on marine ecosystems, and only recently has scientific attention shifted to the effect of plastics in terrestrial ecosystems^3,7^.

The newest results suggest that MNPs may limit nutrient and water uptake affecting plant growth, germination and physiology^1,8,9^. As nanoplastics can be transferred from soil to plant roots and to aboveground tissues, they can be found in epidermal cells, apoplast, xylem, cytoplasm and vacuoles^4,10^. Polystyrene nanoplastics were shown to alter carbon metabolism, amino acid biosynthesis, and plant hormone transduction in wheat hydroponic cultures^11^. The presence of nanoplastics in plant cells may cause oxidative stress, manifested by overproduction of reactive oxygen species (ROS) e.g. superoxide anion radical (O_2_^•−^) and hydrogen peroxide (H_2_O_2_)^12^. Plants have evolved ROS-scavenging systems, consisting of enzymatic and nonenzymatic antioxidants^13^. The main antioxidant enzymes include superoxide dismutase, catalase and peroxidases, and their activities are regulated in response to stress via multiple mechanisms including stress-specific phytohormones, i.e. salicylic acid (SA) and abscisic acid, ABA^14,15^. Homeostasis between cytoplasmic SA and its inactive vacuolar storage form, SA-glucoside regulates defence response to stress^16^. Nonenzymatic antioxidants include phenolic compounds^17^. If not scavenged by antioxidants, ROS can damage cell structures including oxidative deterioration of lipids (lipid peroxidation) eventually leading to cell death^18^. Moreover, ROS may change the DNA methylation pattern, which plays a pivotal role in plant responses to environmental stimuli by adjusting the regulation of gene expression^19^. As DNA methylation plays a crucial role in driving organism functioning, changes in DNA methylation patterns due to stress can lead to developmental abnormalities^20,21^. The involvement of ROS, phytohormones and oxidation of lipids in plant response to MNPs has already been observed ^22,23^, however, in-depth understanding of these processes and their interactions at the level of plant cell is still lacking.

For decades, plant cell suspension cultures have been used as a model system to study the response to various stresses^24^. The advantage of cell suspension cultures over the whole plant studies is the possibility of obtaining homogeneous material with synchronized responses to external stimuli, independent of environmental variation^24^. Due to these features, plant cell suspensions are ideal candidates for studying the effect of polystyrene nanoparticles (PNPs) at the cellular level. Our study aimed to examine the response of plant cell defence mechanisms to PNPs. We used plant cell cultures of two dicots, carrot and tomato (*Daucus carota* L., *Solanum lycopersicum* L.) and two monocots, wheat and barley (*Triticum aestivum* L., *Hordeum vulgare* L.), which are widely cultivated agricultural plants. We measured the activities of enzymes involved in ROS scavenging, and nonenzymatic antioxidants and we estimated potential damage to plant cell structures and functioning via lipid peroxidation and DNA methylation patterns.

## Results

### Hydrogen peroxide and enzyme activities

**Figure 1:**
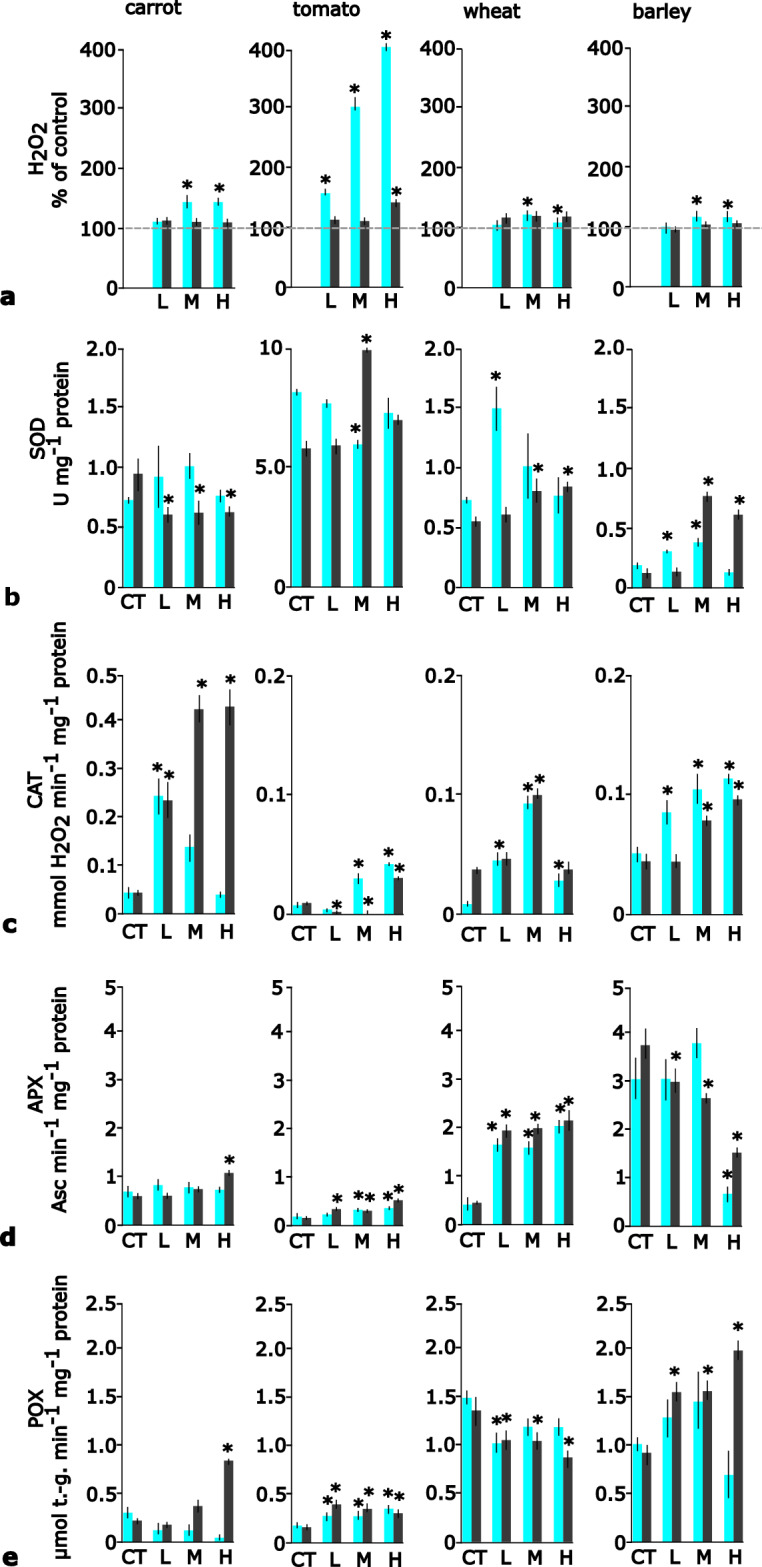
Concentration of H_2_O_2_ and antioxidative enzyme activities in the suspension cultures of carrot, tomato, wheat and barley after addition of polystyrene nanoplastics in low, medium and high concentrations. CT—control, L—low concentration (10^6^ ml^−1^) of polystyrene, M—medium concentration (10^8^ ml^−1^) of polystyrene and H—high concentration (10^10^ ml^−1^) of polystyrene. (**a**) concentration of H_2_O_2_, (**b**) superoxide dismutase, SOD, (**c**) catalase, CAT, (**d**) ascorbate peroxidase, APX, **e**) guaiacol peroxidase, POX. Asc—ascorbate, t.-g. tetraguaiacol. FW—fresh weight. Results presented as the mean and error bars represent standard deviation; results statistically different from controls (p < 0.05, Dunnett’s test) marked with asterisk. 24 h incubation results marked in grey, 96 h in black.

The concentration of H_2_O_2_ in plant cells increased due to PNP additions but the cell response was highly dependent on plant species, plastic concentration, and incubation time (Fig. 1a). The highest H_2_O_2_ concentration (400% of control) was observed in tomato cells after the addition of high PNPs (10^10^ mL^−1^). The concentration of H_2_O_2_ was also elevated after PNP additions in carrot and monocot cells, up to 130% of the control. In all plant cell species, H_2_O_2_ production decreased with time.

Polystyrene nanoplastics induced significant changes in the enzymatic activities of plant cells (Fig. 1b–e). Tomato cells exhibited the highest SOD activities compared to other species (up to 10 times higher), however, significantly higher than those of the control only for medium PNPs (10^8^ mL^−1^) after 96 h (Fig. 1b). For carrot cells SOD activity was similar to that of the control (shorter incubation, 24 h) or lower (longer incubation, 96 h). In general, the activity of SOD in monocot cells increased after the addition of PNPs.

The activity of CAT was the highest in carrot cells, lower in monocot cells and the lowest in tomato (Fig. 1c). Addition of PNPs increased the activity of this enzyme in all species. The pattern of changes in CAT activity was similar in tomato and monocot cells. In carrot cells, CAT activity was highest after longer incubation with PNPs.

The activity of APX was the lowest in tomato, higher in carrot and highest in monocot cells (Fig. 1d). In tomato and barley cells addition of PNPs increased APX activity in a dose-dependent manner. In carrot cells only the highest PNPs caused significantly higher APX activity, and in wheat APX activities were similar or lower that of the control.

The activities of POX were the lowest in tomato and carrot and the highest in monocot cells (Fig. 1e). In all species except wheat, POX activities increased after the addition of PNPs.

### Lipid peroxidation and total phenolic content

**Figure 2:**
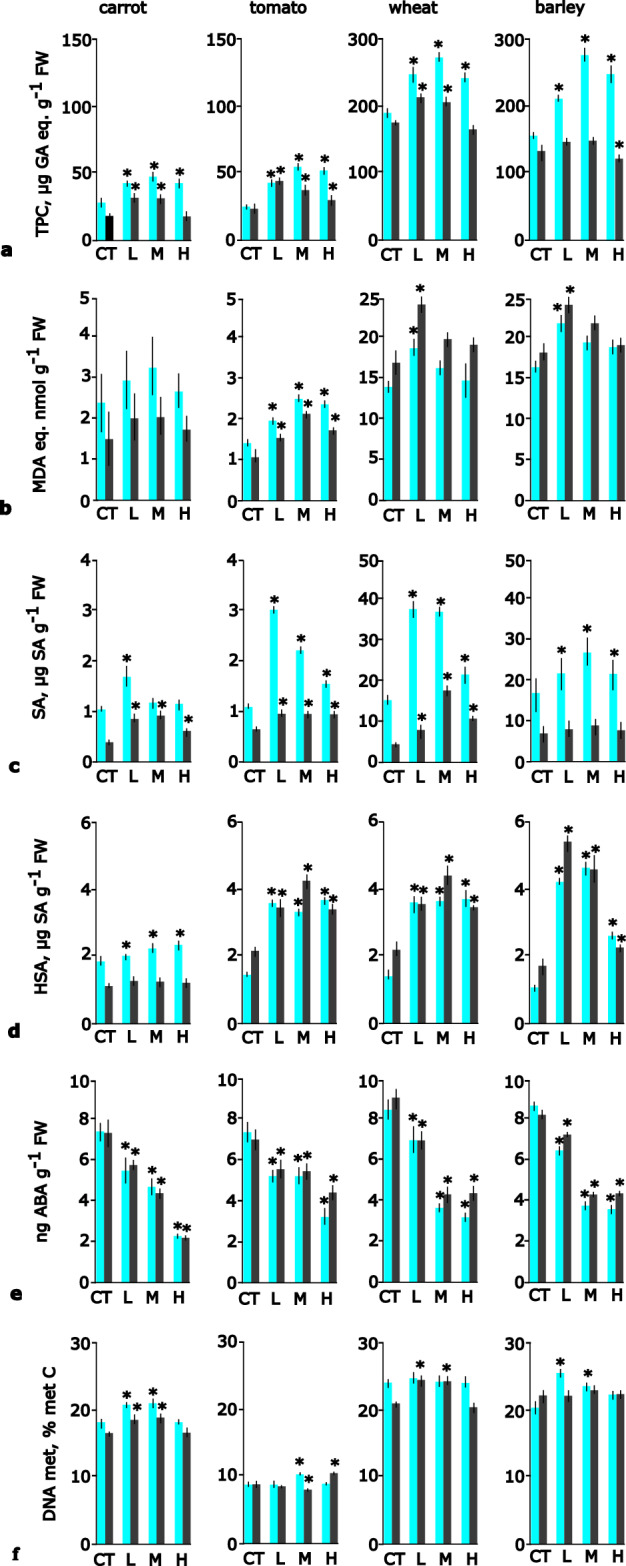
Concentration of total phenolics, lipid peroxidation, concentration of hormones and DNA methylation level in suspension cultures of carrot, tomato, wheat and barley after addition of polystyrene nanoplastics in low, medium and high concentrations. CT—control, L—low concentration (10^6^ ml^−1^) of polystyrene, M—medium concentration (10^8^ ml^−1^) of polystyrene and H—high concentration (10^10^ ml^−1^) of polystyrene. (**a**) total phenolic content, TPC, (**b**) lipid peroxidation (**c**) salicylic acid, SA, (**d**) SA-glucosides, HSA, (**e**) abscisic acid, ABA, (**f**) DNA methylation. FW—fresh weight. TPC—total phenolic content, MDA- malondialdehyde, metC—methylated cytosine. Results presented as the mean and error bars represent standard deviation; results statistically different from controls (p < 0.05, Dunnett’s test) marked with asterisk. 24 h incubation results marked in grey, 96 h in black.

The total phenolic content was 4-times higher in monocot cells than in dicot cells (Fig. 2a). For all species, PNP additions increased to some extent TPC content, especially after a shorter incubation time (24 h). Compared to other species, tomato cells were especially affected by PNPs as TPC was significantly elevated throughout the whole experiment and for all plastic concentrations.

The concentration of MDA was 5-times higher for monocots than in dicot cells (Fig. 2b). In carrot cells, the MDA concentration did not change during the experiment. On the other hand, in tomato cells, the addition of PNPs at all concentrations increased the MDA level. In monocot cells, only low PNPs (10^6^ mL^−1^) increased MDA above the level of the control.

### Plant hormones

The concentration of SA was 10-times higher in monocot cells than in dicot cells, but HSA was at a comparable level (Fig. 2c,d). The addition of PNPs increased the concentration of SA and HSA in the cells. For the two species, wheat and tomato SA and HSA were elevated throughout the experiment, however, in barley cells this pattern was observed mainly for HSA. In carrot cells, the addition of PNPs increased HSA after shorter incubation and SA after longer incubation time.

The concentrations of ABA (Fig. 2e) were low for all species and decreased with increasing concentrations of PNPs. This pattern was linear in dicot cells, and in monocot cells, medium and highest PNPs caused the same decrease in ABA concentration.

### DNA methylation

Overall, the level of DNA methylation was highest in monocot cells, lower in carrot and lowest in tomato cells (Fig. 2f). After the addition of PNPs, DNA methylation in carrot and monocot cells increased for low and medium, but not highest PNPs. However, in tomato cells increased DNA methylation was observed for medium and high PNPs, except for medium PNPs after 96 h.

### Correlations

The concentration of H_2_O_2_ was positively correlated with enzymes scavenging H_2_O_2_ in tomato and wheat cells and barley cells only after a shorter incubation time (see Supplementary Fig. S1-S4). For carrot, there was no correlation between H_2_O_2_ and enzymes scavenging this ROS. Moreover, enzymes scavenging H_2_O_2_ were positively correlated with SA in carrot and wheat cells, in tomato only after a longer incubation time and in barley only after a shorter incubation time.

The concentration of ABA was mostly negatively correlated with enzyme activities, TPC and MDA.

The concentration of MDA was positively correlated with TPC, however, this relationship was significant only in tomato cells (shorter incubation) and wheat (longer incubation). The total phenolic content was positively correlated with SA concentration in both monocots (shorter incubation) and dicots (longer incubation). Moreover, TPC also correlated positively with H_2_O_2_ concentration in tomato cells (shorter incubation) and in carrot and monocot cells (longer incubation).

Methylation of DNA was positively correlated with MDA in monocot cells (shorter incubation).

Combining all plant species with no separation to incubation time provided strong positive correlations between H_2_O_2_ scavenging enzymes (CAT, APX and POX), and negative correlations between SOD activity and DNA methylation. On the other hand, MDA was positively correlated with DNA methylation and TPC with H_2_O_2_ concentration (see Supplemenatry Fig. S5).

Individual data from all four plants separated logically according to time and the PNPs concentration (see Supplementary Figs. S6, S7, S8 and S9). In dicots TPC, SA and HSA cluster together and have a positive correlation with increasing PNPs concentration (see Supplementary Figs. S8, S9). The concentration of ABA increased towards the control treatment. In monocots TPC, and SA cluster closely together but the connection with increasing PNPs concentration is not that clear. Concentrations of ABA and CAT seem to have an opposite effect in all plant species.

## Discussion

Our results highlight that PNPs cause an antioxidative response at the level of plant cells and this response depends on the plant species, time of incubation and plastic concentration. To our knowledge, this is the first time a more holistic view of the effect of PNPs has been provided including ROS, antioxidant mechanisms (enzymes and secondary compounds), lipid peroxidation and hormonal response. Moreover, the effect of PNPs on DNA methylation has been shown up to now only for animals^25^.

After the addition of PNPs plant cells increased H_2_O_2_ production, which has been observed also for whole plants, e.g. onion^22^ and rice^10^. In line with that, due to PNP-caused oxidative stress main ROS scavenging enzymes exerted higher activities, as reflected in literature from whole plants (e.g. castor bean, Jiang et al., 2019). In our study, H_2_O_2_ production was the highest for tomato cells, corresponding with the highest SOD activity catalyzing the dismutation of O_2_^•−^ producing H_2_O_2_^26^. Moreover, the H_2_O_2_ scavenging enzymes, CAT, APX and POX were to some extent lowest in tomato cells, making it possible to sustain a high level of H_2_O_2_. This was accompanied by an increase of lipid peroxidation in tomato cells, which was not clearly observed for other species indicating that tomato is more susceptible to PNPs than other species. Carrot and monocots showed higher activities of ROS scavenging enzymes and lower levels of H_2_O_2_. Thus, the antioxidant response of monocots and carrot was more sufficient to prevent ROS-induced damage resulting in lower lipid peroxidation levels after the addition of PNPs. The pattern of changes in enzyme activities was dependent not only on plant species, but also on incubation time, and PNP concentrations. These changes could be related to various functions of enzymes and their affinities to ROS^27^ and thus different patterns of enzyme response to PNPs. This calls for more studies involving e.g. plant cells overexpressing specific enzymes and with knockout of specific enzymes to disentangle the role of each enzyme in response to plastics.

It is well documented that salicylic acid (SA) plays a central role in stress signaling^13^. In our study SA concentration was elevated in response to PNPs, especially for monocots and tomato, but less for carrot cells. Numerous studies confirmed that SA enables plants to cope with stresses by regulating the antioxidant enzyme activities^28^. On the other hand, increased H_2_O_2_ production may enhance SA accumulation and inhibit CAT activity leading to elevated H_2_O_2_ concentrations^29^. In addition, SA elevates the production of plant secondary metabolites via increasing the activity of phenylalanine ammonia-lyase, a key enzyme in the biosynthesis of phenolic compounds^13^. In fact, we found that the total phenolic content was increased in all studied species, regardless of the PNPs concentration, correlating positively with SA concentration (see Supplementary Figures for all correlations). In our study, all species showed the same pattern of response to PNPs by decreasing the concentration of abscisic acid, another plant hormone involved in reaction to stress^26^. The decrease in ABA level might result from catabolism to phaseic acid, which acts as a signalling molecule in stress tolerance^14^. Interrelations between H_2_O_2_, antioxidant enzymes and hormones point to the concerted response of plant cells to PNPs.

DNA methylation regulates the expression of genes in a complex redox system, however, ROS also affect the epigenetic mechanisms of gene regulation^30^. We found that DNA methylation increased due to PNP additions in all studied plants, corresponding with changes in antioxidative enzyme activities. Similar results were obtained by other researchers^31^ who studied the influence of aluminium stress on maize (*Zea mays* L.) in terms of oxidative damage and variations in DNA methylation patterns. Thus, potential changes in gene expression via DNA methylation represent another route by which PNPs affect on plant cell performance. DNA methylation, though enhanced due to PNPs-induced stress, did not increase in a dose-dependent manner. The reason behind this could be that stress may also activate demethylation, thus alleviating global methylation pattern as shown for drought stress^32^.

All the results taken together (no separation to species or incubation time) unraveled positive correlations between ROS-scavenging enzymes (CAT, APX, POX) and between TPC (non-enzymatic ROS-scavengers) and H_2_O_2_. This underlines that plants not only elevated ROS production due to PNPs but also induced plant response mechanisms. However, when efficiency of defense mechanisms was exceeded, we could observe elevated lipid peroxidation and correlated with it enhanced methylation of DNA.

**Figure 3:**
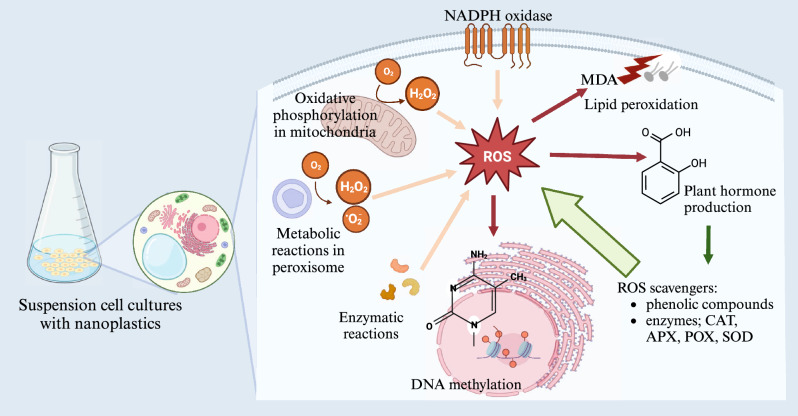
Schematic presentation of experimental design and processes affected by PNPs at the level of the cell. Created with BioRender.com.

In conclusion, our work demonstrates that PNPs contribute to oxidative stress in suspension cell cultures and that the observed changes in the parameters studied were dependent on plant species, exposure time and PNP concentrations (Fig. 3). There was no clear pattern in response to monocots compared to dicots. Although monocots (wheat and barley) usually showed similar responses to each other with often higher activities (CAT, POX, APX) and concentrations (MDA, SA, total phenolic content, DNA methylation level) than tomato, carrot more closely followed the response of monocots than dicot. Due to very high H_2_O_2_, lipid peroxidation and somewhat lower enzyme activities scavenging H_2_O_2_ tomato may be more susceptible to PNPs than monocots or carrot. Subsequent work is needed to deeper understand the potential negative impact of PNPs on plants.

## Materials and methods

### Cell cultures

Cells of carrot (*Daucus carota* ssp. *sativus* L.) cv. Koral were maintained in ZSMI, a modified B5^33^ medium [B5 salts, 500 mg L^−1^ KNO_3_, 250 mg L^−1^ casein hydrolysate, 0.5 mg L^−1^ nicotinic acid, 0.1 mg L^−1^ thiamin, 0.1 mg L^−1^ pyridoxin, 20 g L^−1^ sucrose and 0.4 mg L^−1^ 2,4-D, pH 5.7] in dark at 25 °C. Cells of tomato (*Solanum lycopersicum* L.) cv. Remiz were maintained in modified^34^ medium [MS salts and vitamins, 2 mg L^−1^ glycine, 200 mg L^−1^ L-glutamine, 30 g L^−1^ sucrose, 0.2 mg L^−1^ 6-benzylaminopurine (BAP), 1 mg L^−1^ 2,4-D, pH 5.7] in dark at 25 °C. Cells of wheat (*Triticum aestivum* L.) cv. Svilena and barley (*Hordeum vulgare* L.) cv. Scarlett were maintained in a modified liquid 190–2 medium^35^ supplemented with 1.5 mg L^−1^ biotin, 438 mg L^−1^ L-glutamine, 90 mg L^−1^ maltose and 2 mg L^−1^ 2,4-D in 16/8 h light/dark at 25 °C. Source of cells: barley and wheat were anther cultures, carrot cells were obtained from roots and tomato from segments of hypocotyl and cotyledones. Stage of growth: late expotential phase (24 h) and stationary phase (96 h).

### Polystyrene nanoplastics

We used a solution of PNPs with a diameter of 50 nm (coefficient of variance 15%) and concentration of 3.64 × 10^15^ particles mL^−1^ (Polyscience Polybead® Microspheres, Amsterdam, Netherlands; CAT#08,691–10). Polybead® Microspheres are monodisperse polystyrene microspheres. The solution of PNPs was cleaned by vortexing for 30 s and sonication at 42 kHz for 10 s following centrifugation (5 min, 13000 g) and replacing supernatant with ultrapure water as in^6^. Three PNP concentrations (low 10^6^, medium 10^8^ and high 10^10^ particles mL^−1^) were added to cell suspensions and incubated for 24 h (shorter incubation) and 96 h (longer incubation). Survival of the plant cells was above 90% for both timepoints. Each treatment was replicated 5 times. PNP concentrations were based on Bosker et al. (2019) and adjusted according to a preliminary test (Supplementary Materials Table S3) in which no response to concentrations lower than 10^6^ mL^−1^ was observed.

### H_*2*_O_*2*_production and enzymatic activities

The production of H_2_O_2_ was measured with xylenol orange via the reduction of H_2_O_2_ by ferrous ions forming a ferric product-xylenol orange complex^36^.

For enzyme extraction, 300 mg of plant cultures was homogenized in 1 ml of 50 mM phosphate buffer (pH 7.0) containing 1 mmol L^−1^ EDTA, 1% PVP and 1 mol L^−1^ NaCl and centrifuged (15 min, 5000 g, 4 °C). Extraction for SOD included 1 mmol L^−1^ sodium ascorbate. Catalase (CAT, EC 1.11.1.6) activity was measured in an assay mixture with 50 mmol L^−1^ phosphate buffer (pH 7.0) and 15 mmol L^−1^ H_2_O_2_^37^. Decomposition of H_2_O_2_ was measured at 240 nm, and CAT activity was expressed in μmol H_2_O_2_ min^−1^ mg^−1^ protein. Guaiacol peroxidase (POX, EC 1.11.1.7) activity was measured in an assay mixture containing 10 mmol L^−1^ phosphate buffer, pH 7.0 and 1% guaiacol and 100 mmol L^−1^ H_2_O_2_^38^. The oxidation of guaiacol to tetraguaiacol was measured at 470 nm and POX activity was expressed in µmol of tetraguaiacol min^−1^ mg^−1^ protein. Superoxide dismutase (SOD, EC 1.15.1.1) activity was measured in an assay mixture containing 3 mM EDTA, 73 µmol L^−1^ nitrotetrazolium blue (NBT), 13 mmol L^−1^ methionine in 50 mmol L^−1^ phosphate buffer (pH 6.4) and 60 µmol L^−1^ riboflavin^39^. Samples were placed under a UV lamp for 10 min for superoxide anion generation and the degree of inhibition of NBT reduction to diformazan by O_2_^.−^ was measured at 560 nm. SOD activity was expressed in U mg^−1^ protein. Ascorbate peroxidase (APX, EC 1.11.1.11) activity was measured in an assay mixture containing 50 mmol L^−1^ phosphate buffer (pH 7.0), 1 mmol L^−1^ EDTA, 0.25 mmol L^−1^ sodium ascorbate and 25 μmol L^−1^ H_2_O_2_^40^. The oxidation of ascorbate to dehydroascorbate was measured at 265 nm and APX activity was expressed in µmol ascorbate min^−1^ mg^−1^ protein. Protein content was determined according to previous method^41^.

### Lipid peroxidation and total phenolic content

Lipid peroxidation was measured with thiobarbituric acid (TBA) method^18^; 250 mg of the cell cultures was homogenized with liquid N and with 1 mL 0.1% trichloroacetic acid (TCA), centrifuged and mixed with 20% TCA containing 0.5% TBA, heated at 95 °C for 30 min. The absorbances were read at a microplate reader (BMGLabtech, ClarioStar) at 532 nm and at 600 nm to subtract non-specific absorption and at 440 nm to subtract sucrose. The results are presented as malondialdehyde (MDA) equivalent (nmol g^−1^ fresh weight, FW).

Total phenolic content was measured as described previously^17^; 300 mg of plant cell cultures was ground with liquid N, mixed with 80% methanol, and incubated for 1 h at room temperature. Following centrifugation, 25 µL of supernatant was mixed with 75 µL of water and 25 µL of Folin-Ciocalteu reagent and incubated for 6 min, then 100 µL 7.5% Na_2_CO_3_ was added and incubated in dark for 90 min at room temperature. As a standard gallic acid was used (12.5–200 µg mL^−1^). Absorbance at 765 nm was measured and total phenolic content was expressed as µg gallic acid (GA) equivalent g^−1^ FW.

### Plant hormones

Free salicylic acid (SA) and SA-glucosides (HSA) were quantified by high-pressure liquid chromatography (ARC HPLC, Waters) as described previously^16^. Plant cells (250 mg) were ground with liquid N and extracted with 70% ethanol and re-extracted with 90% methanol. Extracts were combined and methanol and ethanol were evaporated in a vacuum concentrator (Speed Vac 2–18 CDplus, Martin Christ, Osterode am Harz, Germany). Then, 65 µL 20% TCA, and 650 µL of ethyl acetate:cyclohexane were added and the upper phase was moved into a new tube and the water phase was re-extracted. Upper phases were pooled and evaporated, and dry residue was solubilized in 100 µL 10% methanol with 0.1% trifluoroacetic acid (TFA). This fraction represented free SA. Water phases were used to extract SA-glucosides hydrolyzed with 0.3 mL 12 mol L^−1^ HCl and incubated at 80 °C for 1 h. Samples were extracted twice with ethyl acetate:cyclohexane and evaporated in a vacuum concentrator. The dry residue was solubilized in 100 µL 10% methanol with 0.1% TFA. This fraction represented SA-glucosides. Measurements of free and hydrolyzed SA were conducted with HPLC equipped with C18 column (Phenomenex, Torrance, California, United States) and eluted with 10%-82% methanol gradient over 30 min. The Concentration of SA was quantified with a fluorescence detector (excitation 305 nm, emission 407 nm). Anisic acid was used as an internal standard. SA concentrations were expressed as µg SA g^−1^ FW.

Abscisic acid was measured as described before^42^; 500 mg of plant cells were mixed with liquid N and 1.5 mL of 70% methanol and stirred overnight at 4 °C. Methanol was evaporated and 50 µL of borate buffer (pH 8.5) was added and a sample was partitioned with ethyl acetate three times. The ethyl acetate phase was removed, and the water extract was adjusted to pH 2.5 and partitioned with ethyl acetate three times. Ethyl acetate was evaporated, and the residue dissolved in 100 µL methanol. The sample was injected into HPLC (ARC HPLC, Waters, Milford, MA, United States) with Luna C18 column (Phenomenex) eluted with 26% acetonitrile adjusted to pH 3.7 with acetic acid. Detection was done with a UV detector (265 nm).

### DNA methylation

DNA methylation pattern is measured after digestion of extracted and purified DNA with HPLC. This means that after DNA digestion released nucleotides are separated in the HPLC column and cytosine and methylated cytosine are quantified using standards. Plant DNA was extracted and purified with a NucleoSpin Food kit (Macharey-Nagel) and quantified using NanoDrop 100 (Thermo Fisher Scientific, Waltham, United States). DNA methylation was measured as described before^21^; 3 µg of DNA were digested with 3 μL of DNA degradase (Zymo Research Corp) containing 7.5 μL of 10 × DNA degradase reaction buffer for 3 h at 37 °C in an orbital incubator at 100 rpm. Then samples were re-quantified using the NanoDrop, and the volume of each sample was adjusted to 75 μL with nuclease-free water. Samples were run on HPLC (ARC HPLC, Waters, Milford, United States) with Luna C8 column (Phenomenex) eluted with 3% acetonitrile with 0.5% acetic acid and detected with a UV detector at 275 nm.

### Statistics

We used R4.2.2. (R Core Team 2023) with libraries car^43^, multcomp^44^ and corrplot^45^. We used factor analysis of mixed data (FAMD) to explore the associations between both quantitative and qualitative variables with libraries FactoMineR^46^ and factoextra^47^. We conducted two-way-ANOVA with PNP treatments and sampling timepoint and their interactions as explanatory variables. If needed, the variable was transformed to meet the assumptions of normality (Levene’s test). If there was a significant interaction of time and treatment, then treatment effect was calculated for both timepoints separately. As post-hoc we used Dunnett’s test. We adjusted the *p*-values for multiple comparisons with Holms’s method.

### Statement on the collection of plant material

The plant materials used in this study were sourced from controlled cultivation and all collection were made in accordance with institutional, national and international guidelines for the collection of wild plants.

### Supplementary Information

Supplementary Information.

## Data Availability

The datasets used and/or analysed during the current study are available from the corresponding author on reasonable request.
