# Supplementary material for: Polystyrene nanoparticles induce concerted response of plant defense mechanisms in plant cells
Source: Sci Rep. 2023 Dec 16;13:22423. doi: 10.1038/s41598-023-50104-5 (PMC10725457; doi:10.1038/s41598-023-50104-5)
Supplement: Supplementary file 1 — Supplementary Information. [file 41598_2023_50104_MOESM1_ESM.pdf]

Supplementary materials

Title: Polystyrene nanoparticles induce concerted response of plant defense mechanisms in plant cells.

Authors: Sylwia Adamczyk[1]*, Joanna Chojak-Koźniewska[2], Sylwia Oleszczuk[2], Krzysztof Michalski[2], Sannakajsa Velmala[1], Laura J. Zantis[3], Thijs Bosker[3,4], Janusz Zimny[2], Bartosz Adamczyk[1], Slawomir Sowa[2]

Supplementary material - Correlation plots

Suspension culture experiment

Carrot

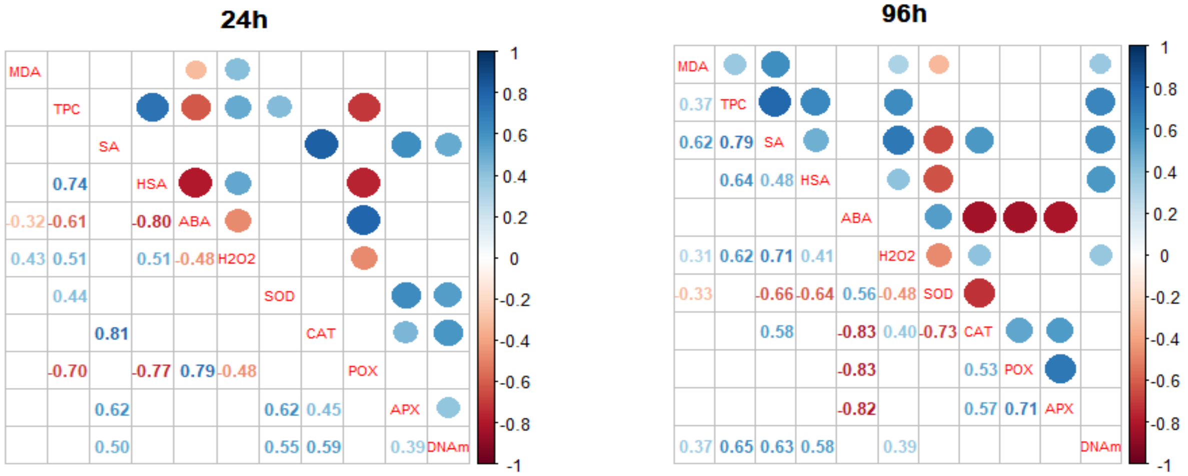

Figure S1. The correlation between H_2O_2, enzymatic and non-enzymatic antioxidants, lipid peroxidation and DNA methylation in carrot suspension culture after 24h and 96h**.** The lower triangle shows the correlation coefficients. Only statistically significant correlations are shown ($P<0.05$). Blue values are showing positive correlations, red ones negative correlations. MDA- malondialdehyde, TPC – total phenolic content, SA – salicylic acid, HSA – hydrolyzed SA, ABA – abscisic acid, SOD – superoxide dismutase, CAT – catalase, POX – guaiacol peroxidase, APX – ascorbate peroxidase, DNAmet – DNA methylation.

Supplementary materials

Tomato

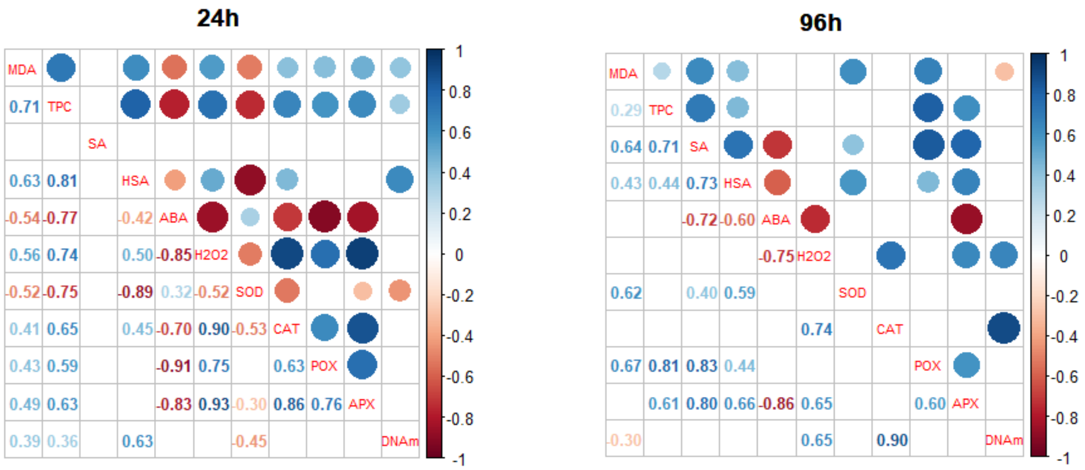

Figure S2. The correlation between H_2O_2, enzymatic and non-enzymatic antioxidants, lipid peroxidation and DNA methylation in tomato suspension culture after 24h and 96h. The lower triangle shows the correlation coefficients. Only statistically significant correlations are shown ($P<0.05$).). Blue values are showing positive correlations, red ones negative correlations. MDA- malondialdehyde, TPC – total phenolic content, SA – salicylic acid, HSA – hydrolyzed SA, ABA – abscisic acid, SOD – superoxide dismutase, CAT – catalase, POX – guaiacol peroxidase, APX – ascorbate peroxidase, DNAmet – DNA methylation.

Supplementary materials

Wheat

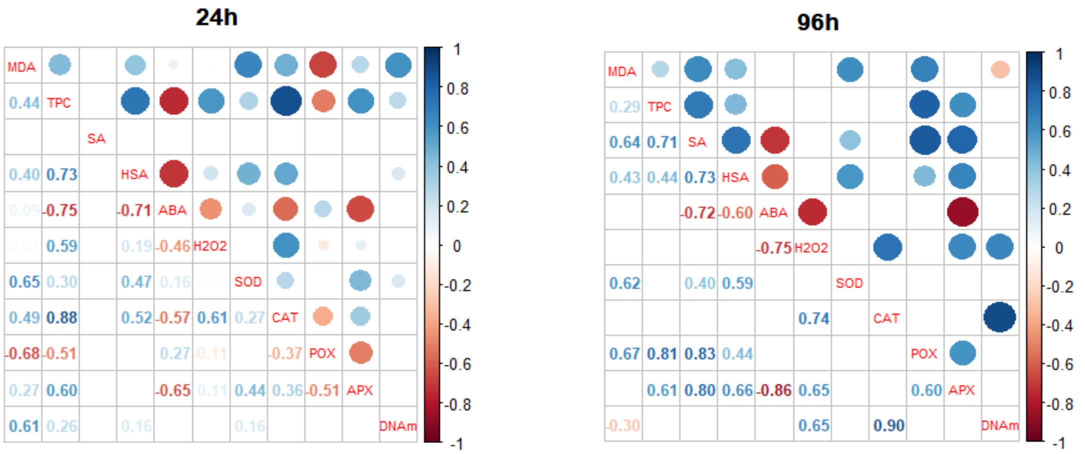

Figure S3. The correlation between H_2O_2, enzymatic and non-enzymatic antioxidants, lipid peroxidation and DNA methylation in wheat suspension culture after 24h and 96h. The lower triangle shows the correlation coefficients. Only statistically significant correlations are shown ($P<0.05$).). Blue values are showing positive correlations, red ones negative correlations. MDA- malondialdehyde, TPC – total phenolic content, SA – salicylic acid, HSA – hydrolyzed SA, ABA – abscisic acid, SOD – superoxide dismutase, CAT – catalase, POX – guaiacol peroxidase, APX – ascorbate peroxidase, DNAmet – DNA methylation.

Supplementary materials

Barley

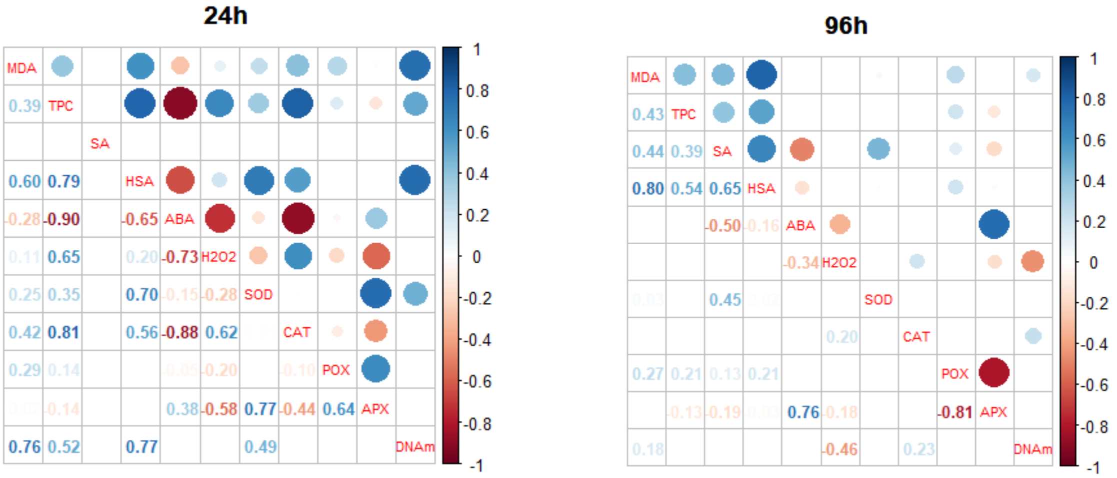

Figure S4. The correlation between H_2O_2, enzymatic and non-enzymatic antioxidants, lipid peroxidation and DNA methylation in barley suspension culture after 24h and 96h. The lower triangle shows the correlation coefficients. Only statistically significant correlations are shown ($P<0.05$).). Blue values are showing positive correlations, red ones negative correlations. MDA- malondialdehyde, TPC – total phenolic content, SA – salicylic acid, HSA – hydrolyzed SA, ABA – abscisic acid, SOD – superoxide dismutase, CAT – catalase, POX – guaiacol peroxidase, APX – ascorbate peroxidase, DNAmet – DNA methylation.

All plant species together, no separation according to time

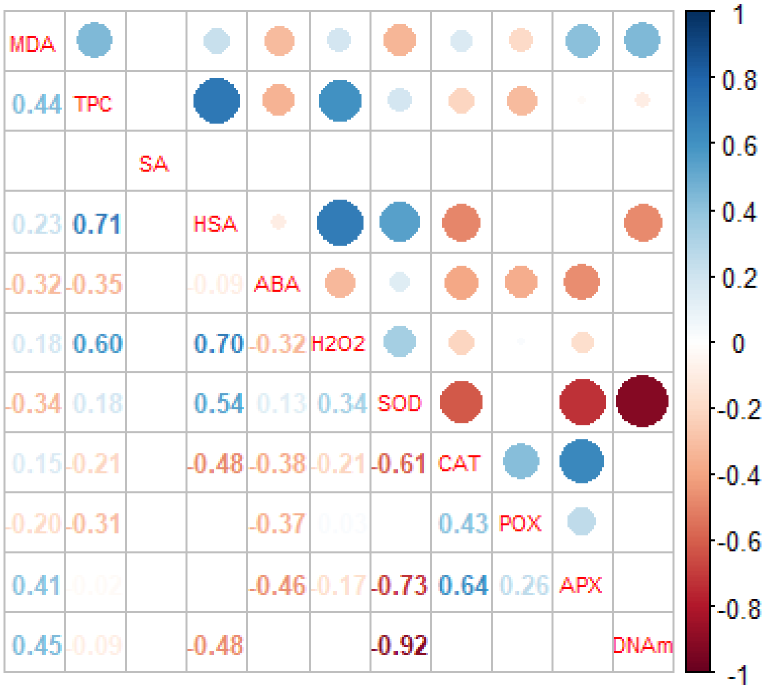

Figure S5. The correlation between H_2O_2, enzymatic and non-enzymatic antioxidants, lipid peroxidation and DNA methylation in all plants together, no separation according to time. The lower triangle shows the correlation coefficients. Only statistically significant correlations are shown ($P<0.05$). Blue values are showing positive correlations, red ones negative correlations. MDA- malondialdehyde, TPC – total phenolic content, SA – salicylic acid, HSA – hydrolyzed SA, ABA – abscisic acid, SOD – superoxide dismutase, CAT – catalase, POX – guaiacol peroxidase, APX – ascorbate peroxidase, DNAmet – DNA methylation.

Supplementary materials

carrot

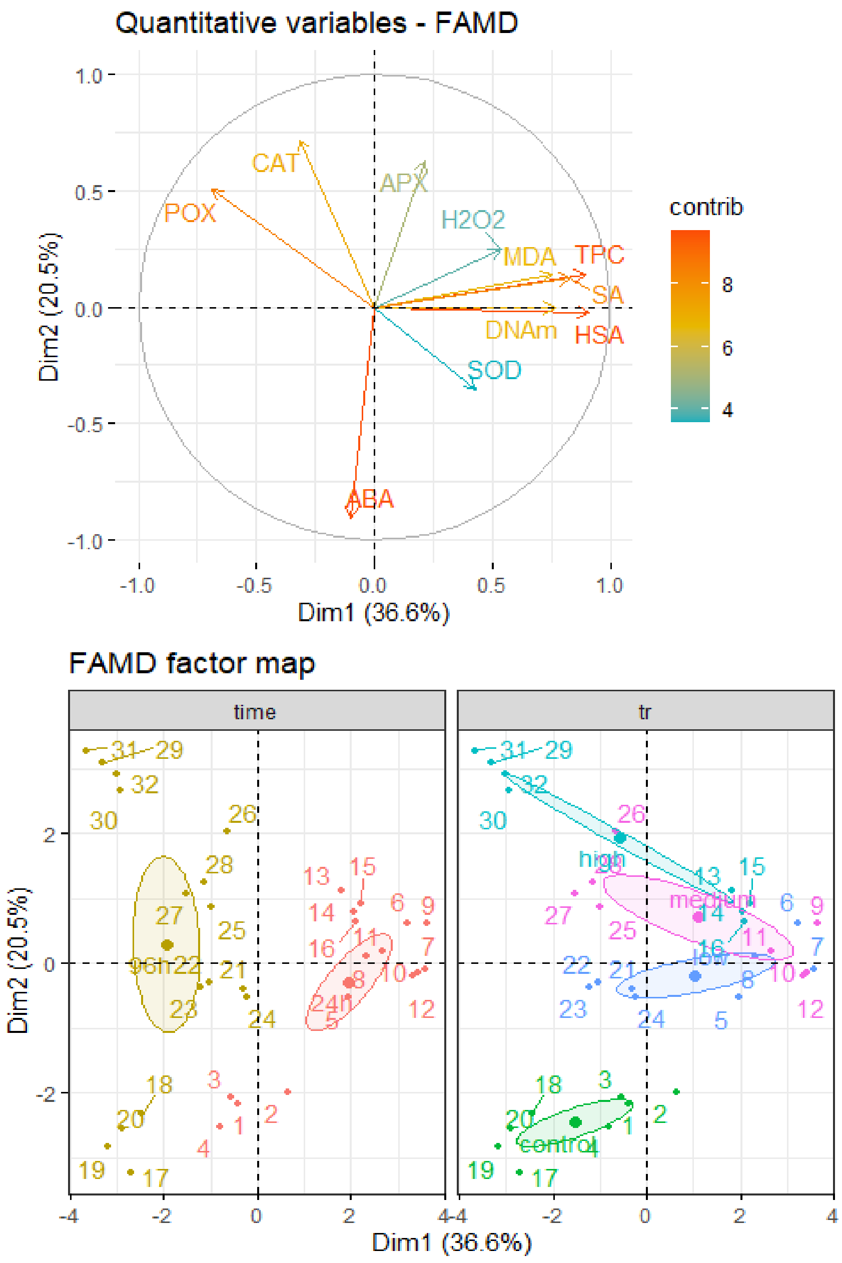

Fig. S6. The correlation circle shows the relationship between quantitative variables, the quality of the representation of variables, as well as the correlation between variables and the dimensions. The most contributing quantitative variables are highlighted with dark orange color. On the factor map individuals with similar profiles are close to each other on the factor map, colored according to the representative categorical variables, here time and plastic concentrations.

Supplementary materials

tomato

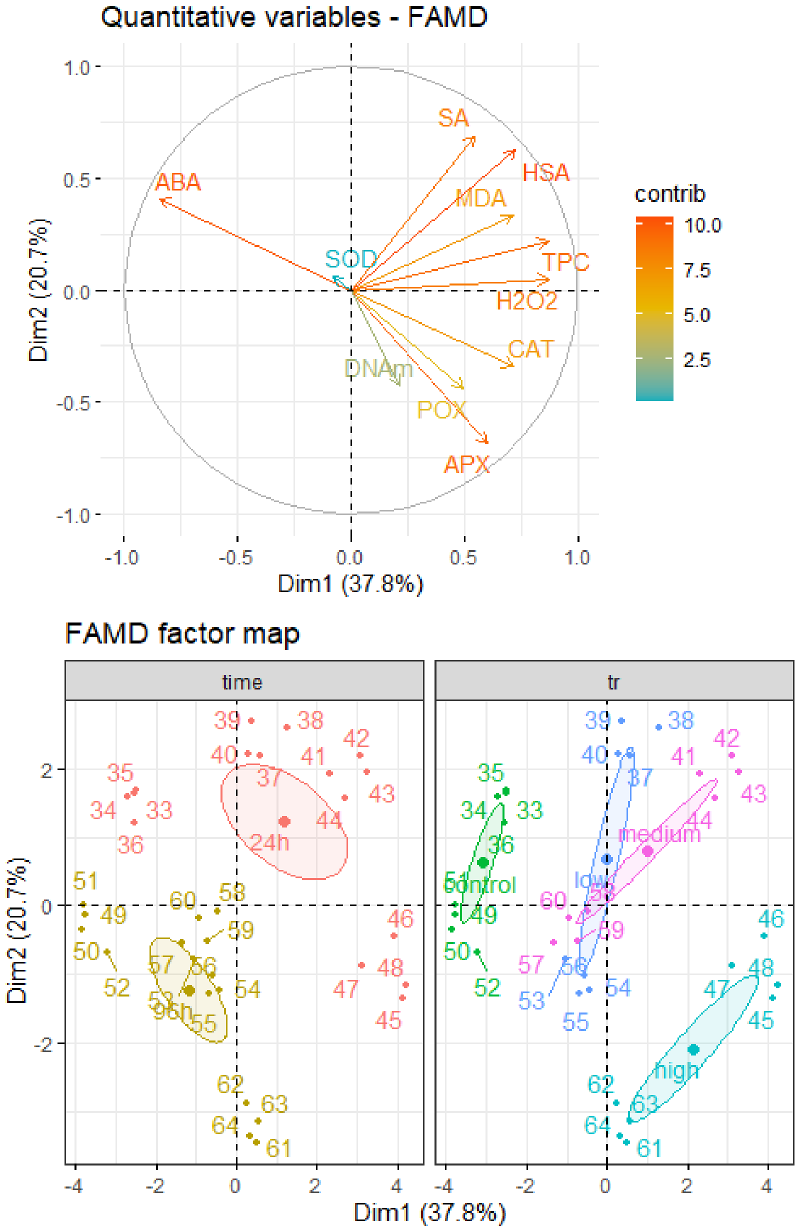

Fig. S7. The correlation circle shows the relationship between quantitative variables, the quality of the representation of variables, as well as the correlation between variables and the dimensions. The most contributing quantitative variables are highlighted with dark orange color. On the factor map individuals with similar profiles are close to each other on the factor map, colored according to the representative categorical variables, here time and plastic concentrations.

barley

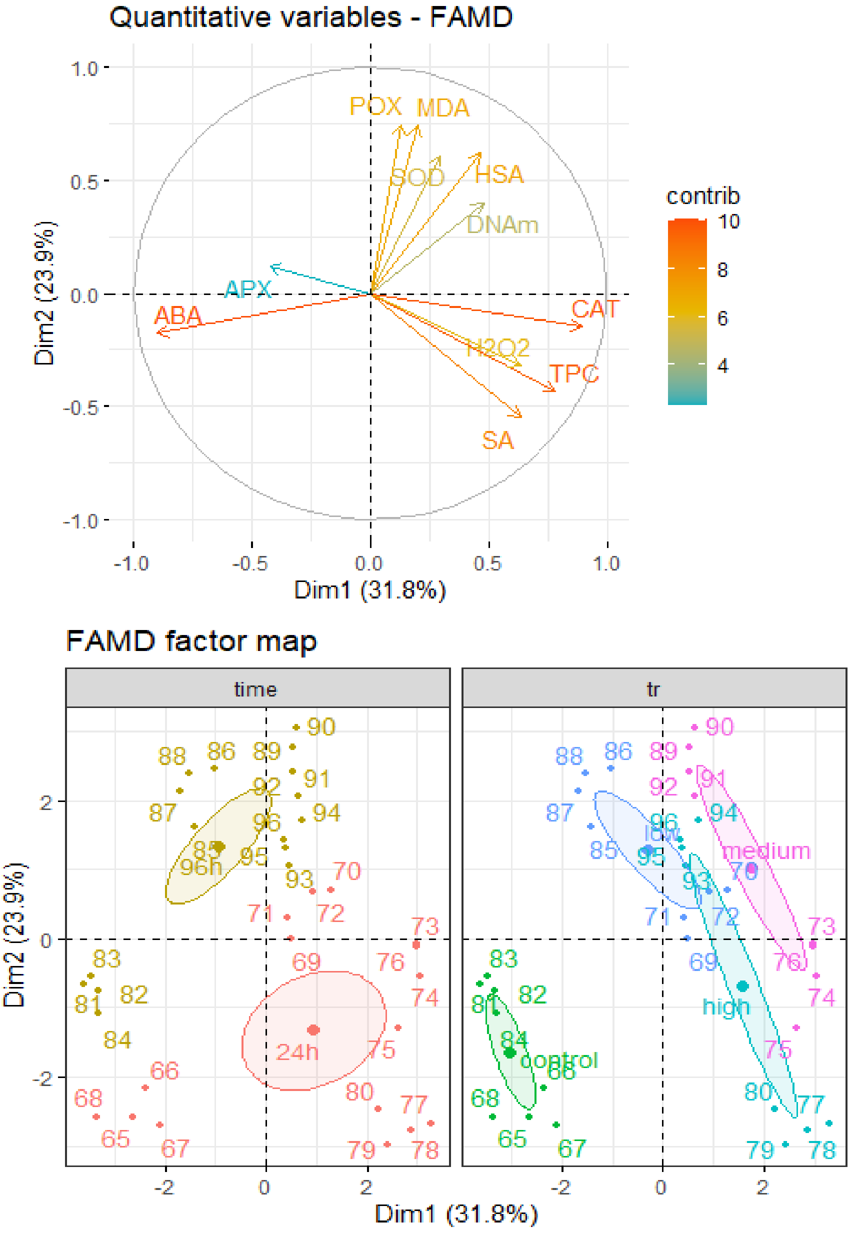

Fig. S8. The correlation circle shows the relationship between quantitative variables, the quality of the representation of variables, as well as the correlation between variables and the dimensions. The most contributing quantitative variables are highlighted with dark orange color. On the factor map individuals with similar profiles are close to each other on the factor map, colored according to the representative categorical variables, here time and plastic concentrations.

Supplementary materials

wheat

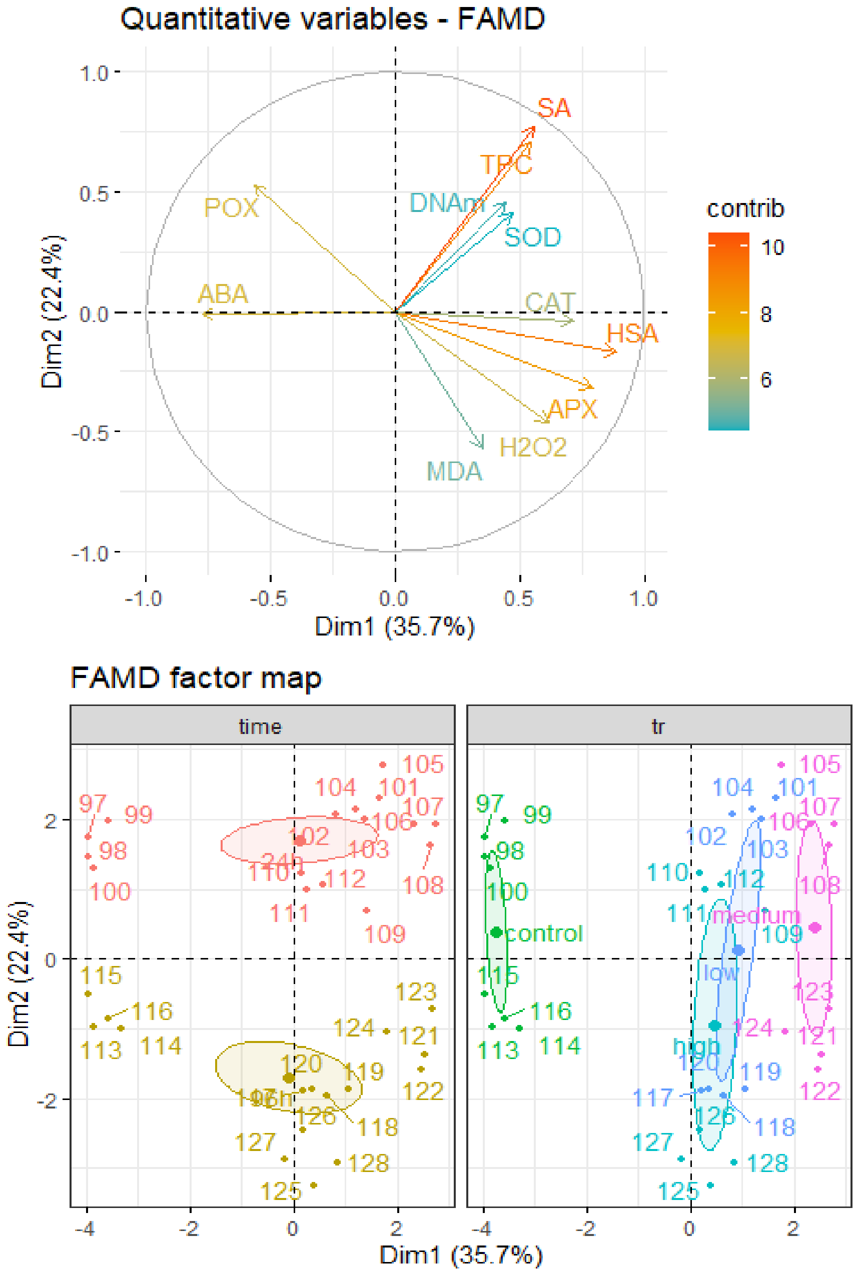

Fig. S9. The correlation circle shows the relationship between quantitative variables, the quality of the representation of variables, as well as the correlation between variables and the dimensions. The most contributing quantitative variables are highlighted with dark orange color. On the factor map individuals with similar profiles are close to each other on the factor map, colored according to the representative categorical variables, here time and plastic concentrations.

Supplementary materials

Table S1. Soluble protein content studied with classic Bradford method. Results presented as mean (µg mL^{-1}) and standard deviation in brackets. No statistically significant differences found.

species	time	control	low	medium	high
Barley	24h	934(271)	890(180)	997(200)	726(122)
	96h	779(163)	911(126)	997(200)	891(73)
wheat	24h	668(82)	772(68)	658(84)	686(40)
	96h	791(106)	758(134)	657(88)	796(84)
carrot	24h	812(222)	481(168)	508(145)	791(86)
	96h	400(168)	379(101)	305(75)	241(22)
tomato	24h	423(45)	438(68)	342(69)	399(86)
	96h	688(134)	645(56)	661(163)	585(32)

Table S2. Total soluble sugar concentrations (mg g^{-1}). Sugars were extracted as described in Zivanovic et al. (2020) and measured on ARC HPLC (Waters) with Rezex column (Phenomenex) eluted with water and concentrations were detected using UV detector (190nm). No statistically significant differences found.

species	time	control	low	medium	high
Barley	24h	42(5.9)	45(3.9)	44(4.5)	38(7.8)
	96h	69(13.1)	80(9.9)	75(8.2)	68(12.1)
wheat	24h	40(8.0)	47(8.3)	45(5.3)	40(6.1)
	96h	82(10.0)	90(9.2)	78(13)	69(9.5)
carrot	24h	50(5.8)	55(7.8)	48(9.2)	46(7.3)
	96h	75(8.8)	85(8.5)	80(5.9)	77(9.9)
tomato	24h	43(9.0)	48(6.9)	48(6.5)	38(4.8)
	96h	48(4.7)	52(9.9)	50(8.7)	45(7.7)

Supplementary materials

Table S3. Preliminary results for effect of PNPs in concentration of 10^4 mL^{-1} on barley cell cultures. Results presented as mean and standard deviation in brackets.

**Barley**	**treatment**	**24h**	**96h**
protein	control	975(209)	902(43)
	10^4	902(43)	758(62)
APX	control	2.15(0.3)	2.64(0.2)
	10^4	2.31(0.3)	2.63(0.5)
CAT	control	0.056(0.013)	0.04(0.01)
	10^4	0.067(0.006)	0.03(0.02)
POX	control	1.24(0.15)	1.52(0.09)
	10^4	2.34(0.4)	1.53(0.12)
SOD	control	0.15(0.05)	0.13(0.008)
	10^4	0.13(0.02)	0.12(0.020)

References

Živanović, B.; Milić Komić, S.; Tosti, T.; Vidović, M.; Prokić, L.; Veljović Jovanović, S. Leaf Soluble Sugars and Free Amino Acids as Important Components of Abscisic Acid—Mediated Drought Response in Tomato. *Plants* 2020, *9*, 1147. https://doi.org/10.3390/plants9091147
